# Sounds Like Respect. The Impact of Background Music on the Acceptance of Gay Men in Audio-Visual Advertising

**DOI:** 10.3389/fpsyg.2021.645533

**Published:** 2021-04-22

**Authors:** Ann-Kristin Herget, Franziska Bötzl

**Affiliations:** Institute Human-Computer-Media, University of Wuerzburg, Wuerzburg, Germany

**Keywords:** music, perception, advertising, musical stereotypes, acceptance of gay men

## Abstract

Companies increasingly seek to use gay protagonists in audio-visual commercials to attract a new affluent target group. There is also growing demand for the diversity present in society to be reflected in media formats such as advertising. Studies have shown, however, that heterosexual consumers (especially men), who may be part of the company's loyal consumer base, tend to react negatively to gay-themed advertising campaigns. Searching for an instrument to mitigate this unwanted effect, the present study investigated whether carefully selected background music can shape the perceived gender of gay male advertising protagonists. In a 2 × 2 between-subjects online experiment (musical connotation × gender of the participant), 218 heterosexual participants watched a commercial promoting engagement rings that featured gay male protagonists, scored with feminine- or masculine-connoted background music. As expected, women generally reacted more positively than men to the advertising. Men exposed to the masculine-connoted background music rated the promoted brand more positively, and masculine music also enhanced (at least in the short term) these men's acceptance of gay men in general (low and medium effect sizes) more than was the case for feminine background music. Carefully selected background music affecting the perceived gender of gay male advertising protagonists may prevent negative reactions from heterosexual audiences and, therefore, motivate companies to use gay protagonists in television commercials on a more regular basis.

## Introduction

There are at least two good reasons why companies increasingly use gay^1^ protagonists (or other members of sexual and gender minorities) in audio-visual commercials. First, companies want to attract a new affluent target group. Gay consumers are considered a “dream market” or a “gold mine” (Um, 2014, p. 812; see also Dotson et al., 2009; Angelini and Bradley, 2010). This group is regarded as having considerably more spending power than other consumer groups, and, to date, they have not been oversaturated with advertising campaigns targeted explicitly toward them (Oakenfull et al., 2008). Second, there is a growing demand for the diversity found in society to be reflected in media formats such as advertising (Dotson et al., 2009). Åkestam et al. (2017), for example, argued that the motivation for the increasing portrayals of gay people in advertising is not only reaching this target group but also generating social change. As a result, the amount of gay-themed advertising appearing in mainstream media is increasing (Grau and Zotos, 2016).

### Attitudes Toward Advertising Featuring Gay Protagonists

Although gay consumers might react positively to advertising portraying gay protagonists (Smith and Malone, 2003; Tuten, 2005; Dotson et al., 2009; Bond and Farrell, 2020b), there is also a downside for companies targeting this “dream market.” Studies have shown that heterosexual consumers—especially men—tend to rate commercials with gay protagonists, as well as the advertised product/brand, more negatively, compared with commercials without gay protagonists (Oakenfull and Greenlee, 2005; Dotson et al., 2009; Um, 2014). The presence of gay protagonists also decreases these consumers' intentions to buy or recommend the advertised products (Hooten et al., 2009; Um, 2016). More explicit portrayals of sexual orientation (e.g., two protagonists embracing vs. two protagonists kissing) correspond to more negative reactions from heterosexual consumers, particularly among heterosexual men (Oakenfull et al., 2008; Dotson et al., 2009; Um, 2016). Although recent research has pointed to slightly more positive tendencies (e.g., Pounders and Mabry-Flynn, 2016; Åkestam et al., 2017; Bond and Farrell, 2020a,b), the literature suggests that heterosexual male consumers have a particular aversion to advertising with gay men as protagonists.

### Social Identity Theory as a Possible Explanation for the Rejection of Gay-Themed Advertising

Consumers' differing reactions toward advertising featuring gay protagonists are often explained using social identity theory (Oakenfull and Greenlee, 2005; Hester and Gibson, 2007; Angelini and Bradley, 2010; Um, 2014; Gong, 2020). According to this theory, every individual defines himself or herself as part of a specific social group. The focus of this process is not only self-identification with members of the in-group who are categorized as similar-looking or like-minded; making a distinction between oneself and individuals considered to belong to out-groups also enhances the group's perceived social standing and every in-group member's self-esteem (Tajfel and Turner, 1986).

Although gay consumers can relate to gay protagonists in commercials as a portrayal of their in-group members (Bhat et al., 1998), heterosexual consumers (especially men) are expected to respond differently. A variety of factors, such as the heterosexist foundations of modern society (Hooghe et al., 2010), lead to heterosexual men being more likely than women to identify with traditional gender-role beliefs. Heterosexual men also tend to rate heterosexuality as an essential factor in their masculinity (Herek, 1984). Vandello et al. (2008) explain this conformity to traditional male gender roles and the avoidance of stereotypically female appearances and behavior based on the precarious manhood theory. In contrast to heterosexual women, heterosexual men tend to perceive their gender identity as a concept that has to be gained—and reaffirmed in case of threats (Glick et al., 2007). Following social identity theory, heterosexual men can be expected to perceive gay people, and especially gay men, as violating traditional gender roles and to see gay men as lacking in masculinity. As a result of the perceived difference between themselves and gay people, heterosexual men, compared with heterosexual women, feel more social pressure to distance themselves from gay people—especially gay men (LaMar and Kite, 1998). This social pressure may explain why heterosexual men evaluate gay-themed advertising more negatively than do heterosexual women and why more explicit portrayals of gay sexual orientation (especially of men) correspond to more negative evaluations. Although companies are keen to tap into the LGBTQ^2^ market, they do not want to offend or lose their loyal majority-heterosexual consumer base through these campaigns (Um, 2014, 2016; Bond and Farrell, 2020a). At this point, the music comes into play.

### Background Music in Audio-Visual Media Formats as an Instrument to Convey Meaning

Under specific circumstances, instrumental background music in audio-visual media formats can activate particular schemata conveying extra-musical meaning (e.g., Boltz, 2001; Shevy, 2007). Musical stereotypes such as specific instruments, musical genres, and the positive or negative emotional connotation of the music can trigger supra-individual associations, which are projected onto the audio-visual media format (e.g., Shevy, 2007, 2008; Wingstedt et al., 2007; Tan, 2017). Accordingly, this schema activation can change the perception and interpretation of the media format's general atmosphere, plot, or protagonists (e.g., Boltz, 2001; Tan et al., 2017; Steffens, 2020; Herget, 2021). In the context of film, for example, Boltz (2001) has shown that different background music can change how viewers judge the inherent character and temperament of protagonists in ambiguous short films. The schema-activating potential of music has also been shown in advertising. Martín-Santana et al. (2015) found that varying the background music affected the credibility of spokespersons in a radio commercial, and Ziv et al. (2012) investigated music's influence in commercials in terms of promoting unethical behavior (see also Hung, 2000, 2001; Shevy and Kristen, 2011; Oakes and North, 2014). Some studies have even indicated that a predictable positive or negative attitude change regarding the media format's subject is induced by positive- or negative-connoted background music tracks (Costabile and Terman, 2013; Nosal et al., 2016).

Which types of background music-induced schema activation are relevant for influencing perceptions of gay male protagonists in audio-visual advertising? How individuals associate certain musical instruments and musical genres with gender has been studied since the 1970s. It is not only adults, but even young children, who align specific musical instruments or musical genres with prominent gender stereotypes (e.g., Marshall and Shibazaki, 2011; Stronsick et al., 2018). Whereas electric guitars and rock music are perceived as more masculine, violins, flutes, and romantic classical music are typically classified as feminine. Therefore, music can (a) evoke specific gender stereotypes and (b) influence the perception and interpretation of a media format and its protagonists. This study attempted to combine these two functions of background music to improve the perception of gay advertising protagonists.

### Hypotheses

Reactions to gay male protagonists in commercials tend to be more negative (and, therefore, more problematic) than reactions to lesbians (e.g., Oakenfull and Greenlee, 2005). Hence, this study concentrated on factors that potentially have a positive influence on the perception of gay male advertising protagonists. Combining theoretical insight and previous research on gay-themed advertising and the effects of background music in audio-visual media formats, the following hypotheses were derived.

Gay male advertising protagonists will be perceived as more masculine if accompanied by masculine-connoted background music, and they will be perceived as more feminine when feminine-connoted music is used. Previous studies on music's potential to alter the recipients' perception of different media formats did not indicate potential gender differences regarding this general music effect **(H1: Gender perception of the protagonist)**. Bearing in mind that heterosexual male consumers dislike gay advertising protagonists because they perceive these gay protagonists as different and less masculine compared with themselves, their evaluation of the brand that these protagonists advertise should be more positive when the protagonists are accompanied by masculine music. Heterosexual women will rate the brand more positively than men (e.g., Oakenfull et al., 2008; Um, 2014), regardless of whether the music is feminine or masculine **(H2: Attitude toward the brand)**. In films and documentaries, specific music can change the recipients' attitudes toward a given subject in predictable ways (e.g., Costabile and Terman, 2013). In a study by Iacoviello et al. (2020), heterosexual men described more negative attitudes toward gay people when confronted with men's feminization (i.e., a perceived threat or challenge of traditional norms of masculinity). A feminine-connoted background music in a commercial with gay male protagonists could be perceived as enhancing men's feminization, while a masculine-connoted music could lessen this masculinity threat. Therefore, this study hypothesized that, depending on whether the advertising background music is masculine- or feminine-connoted, heterosexual men will show more or less tolerance toward gay men in general after watching the commercial. Heterosexual women will be more tolerant of gay men than heterosexual men will be, regardless of whether the background music in their commercial is feminine or masculine **(H3: General acceptance of gay men)**.

## Method

### Selection and Construction of Stimuli

#### Selection of the Advertising Stimulus

To ensure the study's ecological validity, a pre-existing 1 min television commercial produced by the jewelry brand Tiffany, in which a gay man proposes to his boyfriend, was selected as the media stimulus. The commercial has previously been rated as a positive example of commercials portraying gay protagonists (Federici and Bernardelli, 2018). In this study, the commercial's original soundtrack was deleted, and, as the planned manipulation, the commercial was set to masculine- or feminine-connoted instrumental background music.

#### Selection of the Music Stimuli

Specific instruments and certain musical genres can evoke gender stereotypes (e.g., Shevy, 2008; Marshall and Shibazaki, 2011; Herget et al., 2018). On the basis of prominent stereotypes identified in previous research, instrumental rock music with salient electric guitars and drums was selected for the masculine-connoted music condition, and instrumental romantic classical music featuring violins and/or flutes was selected to trigger feminine associations. Since music known to the recipients can sometimes trigger unpredictable associations, we used professional production music libraries as sources for the (unknown) background music versions. Because not every rock music track with electric guitars stimulates associations with masculinity, specific semantic features were also considered in the selection process. For the masculine music condition, music versions classifiable as “strong,” “hard,” and “active” were preferred, whereas the feminine music tracks were “soft,” “tender,” and “smooth” (e.g., Tagg, 2006).

When selecting background music for use in advertising, another factor—“musical fit” (e.g., North et al., 2004, p. 1675)—has to be considered. Music that is intuitively perceived as fitting a commercial can improve the evaluation of the commercial, the advertised product, and the brand (e.g., North et al., 2004; Lavack et al., 2008; Galan, 2009), as well as strengthening the recipient's intention to recommend or buy the product (e.g., North et al., 2004; Lalwani et al., 2009; Park et al., 2014; Herget et al., 2020). Thus, in experiments on music's influence on specific advertising parameters, all selected music versions should generally be perceived as congruent with the commercial. Simultaneously, the music versions should be equally well fitting so that different evaluations of advertising parameters can be attributed to the actual experimental manipulation (in this case, the music's potential to convey different gender stereotypes) and not to the confounding effect of differences between music versions in the degree of fit. To ensure the important impression of realistic commercials (Guido et al., 2016), every music version was edited professionally and structurally congruent onto the advertising stimulus.

Multiple stimulus versions per experimental condition were created to increase the likelihood that the stimulus versions would convey the desired gender association and simultaneously vary as little as possible in terms of musical fit. A pretest was conducted to identify the stimulus versions most suitable for the experiment.

#### Pretest of the Stimuli

In a one-way between-subjects online experiment, 61 participants (79% female, age: *M* = 25.25, *SD* = 7.33) viewed the Tiffany commercial with either the masculine-connoted or the feminine-connoted background music (three feminine and two masculine versions). The participants' perceptions of the protagonists' gender (three items on a five-point Likert scale; based on Grohmann, 2009; α = 0.83, *M* = 2.13, *SD* = 0.94; e.g., “sensitive” (reverse-coded)) and the perceived musical fit (three items on a five-point Likert scale; based on Kellaris et al., 1993; α = 0.85, *M* = 3.28, *SD* = 1.20; e.g., “Regardless of how much I liked or disliked the music, it did seem appropriate for this ad”) were measured. For two stimuli, the feminine- and masculine-connoted music versions actually influenced the participants' perceptions of the protagonists' masculinity (feminine stimulus: *M* = 1.69, *SD* = 0.59; masculine stimulus: *M* = 2.82, *SD* = 1.11). However, the feminine music version was perceived as more congruent (*M* = 4.08, *SD* = 0.81) compared with the masculine version (*M* = 2.87, *SD* = 1.24)—an unplanned difference. In the context of a commercial for engagement rings, any romantic music track would probably be evaluated as more congruent than any powerful music track. Although the feminine music version was perceived as more fitting, both versions (available from the first author upon request) were selected for the experiment despite a possible difference in their degree of musical fit.

#### Experimental Design, Sample, and Measures

A 2 × 2 between-subjects online experiment with experimental manipulation of the background music's masculinity/femininity and a quasi-experimental manipulation of the participant's gender was conducted via the online survey software UNIPARK. A medium effect size of at least *f* ≥ 0.25 (Cohen, 2013) was used as the basis of an a priori sample size calculation. G^*^Power (Faul et al., 2007) indicated a required sample size of *N* = 210 (ANOVA: fixed effects, special, main effects, and interactions, α = 0.05, 1 – β = 0.95, numerator *df* = 1, number of groups = 4). In total, 218 German heterosexual cisgender subjects (73% female, age: *M* = 31.93, *SD* = 12.36) participated in the study. Because this study focused especially on the reactions of heterosexual consumers to gay-themed advertising, the participants were screened by their sexual orientation (a lack of differentiation on this variable was discussed as a study limitation in Angelini and Bradley, 2010, p. 499). Only participants describing themselves as heterosexual, cisgender women or men were included in the final data analysis. Participants who were familiar with the commercial before the study and/or did not complete the online questionnaire with sufficient concentration were also excluded (*n* = 84, dataset: https://tinyurl.com/259c9xfr). The participants were randomly assigned to view the advertising stimulus version with the feminine- or masculine-connoted background music. The participants' perceptions of the protagonists' gender (four items on a five-point Likert scale; α = 0.67, *M* = 2.05, *SD* = 0.71; e.g., “adventurous”) and of the musical fit (four items on a five-point Likert scale; α = 0.93, *M* = 3.58, *SD* = 1.17) were measured as described above for the pretest. The participants also rated the advertised brand (four items on a five-point semantic differential scale; following Spears and Singh, 2004; α = 0.91, *M* = 3.84, *SD* = 0.87; e.g., “negative–positive”) and their general acceptance of gay men [four items on a five-point Likert scale; following Herek, 1984; α = 0.88, *M* = 4.63, *SD* = 0.74, e.g., “I think gay men are disgusting” (reverse-coded)]. The variables were measured in an order least likely to bias results. Participants were invited to a study comparing advertising effects of commercials with male versus female actors to make the study's true purpose less obvious. Questions regarding the background music (perceived musical fit) were grouped with three cover items on the perceived fit of the actors (“Regardless of how much I liked or disliked the actors, they did seem appropriate for this ad”). For more details, please see the questionnaires of the pretest and main experiment in the supplemental online material.

## Results

As in the pretest, the experimental conditions differed regarding the musical fit of the masculine and feminine music versions: The more feminine-connoted music was perceived as significantly more congruent (*M* = 4.16, *SD* = 0.85) than was the masculine-connoted music (*M* = 2.87, *SD* = 1.11), *F*_(1, 216)_ = 95.53, *p* < 0.001, η^2^ = 0.307. Therefore, the potential of a confounding effect of the stimulus versions' different degrees of musical fit on the advertising efficiency had to be considered, especially when assessing the evidence regarding **H2**.

The perceived gender of the gay male advertising protagonists (**H1**) was influenced as expected. Surprisingly, the male advertising protagonists were, in general, perceived as rather feminine. However, participants who watched the stimulus version with masculine-connoted music considered the protagonists as more neutral (*M* = 2.30, *SD* = 0.69), whereas, for those exposed to the feminine-connoted music version, the perception was clearly feminine (*M* = 1.86, *SD* = 0.66), *F*_(1, 216)_ = 23.04, *p* < 0.001, and η^2^ = 0.096.

**Figure 1:**
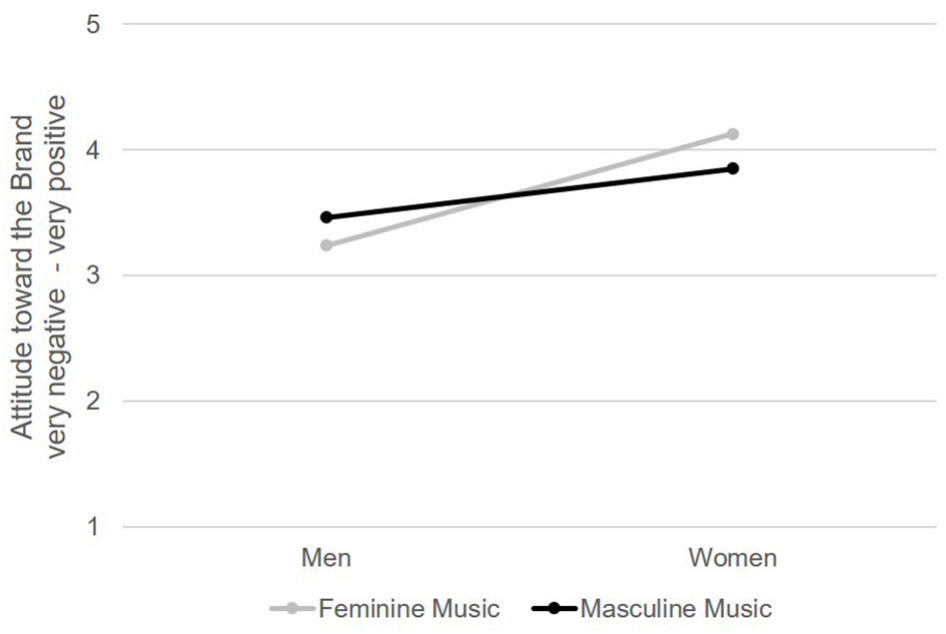
Attitude toward the brand. Attitude toward the brand was measured with four items on a five-point semantic differential scale, e.g., “negative–positive,” *N* = 218.

Regarding attitudes toward the brand (**H2**), heterosexual women, as expected, rated the commercial's brand significantly more positively than did heterosexual men—*F*_(1, 214)_ = 26.87, *p* < 0.001, η^2^ = 0.112 (see Figure 1). As expected, we found no significant main effect for music's connotation, *F*_(1, 214)_ = 0.04, *p* = 0.84, η^2^ < 0.001. Although this difference was not significant either, men listening to masculine music in the commercial liked the brand more than did men who watched the commercial with feminine music—gender × music condition: *F*_(1, 214)_ = 3.88, *p* = 0.05, η^2^ = 0.018 (for more details, see Table 1).

**Table 1:** Influence of the participant's gender and the music's connotation on attitude toward the brand and the general acceptance of Gay Men.

**Construct**	**Participant's gender**	**Music's connotation**	***n***	***M***	***SD***	**95% CI [LL, UL]**
Attitude toward the brand	Men	Feminine	28	3.24	0.86	[2.91, 3.58]
		Masculine	31	3.46	0.78	[3.17, 3.75]
	Women	Feminine	92	4.13	0.81	[3.96, 4.30]
		Masculine	67	3.85	0.79	[3.66, 4.05]
General acceptance of gay men	Men	Feminine	28	3.94	1.14	[3.50, 4.38]
		Masculine	31	4.52	0.71	[4.26, 4.78]
	Women	Feminine	92	4.80	0.48	[4.70, 4.90]
		Masculine	67	4.72	0.67	[4.55, 4.88]

*Descriptive data on the ANOVAs assessing the influence of the participant's gender and the feminine/masculine musical connotation on attitude toward the brand and the general acceptance of gay men. N = 218. Values in square brackets indicate the 95% confidence interval for each mean. LL and UL indicate the lower limit and upper limit of the confidence interval, respectively*.

**Figure 2:**
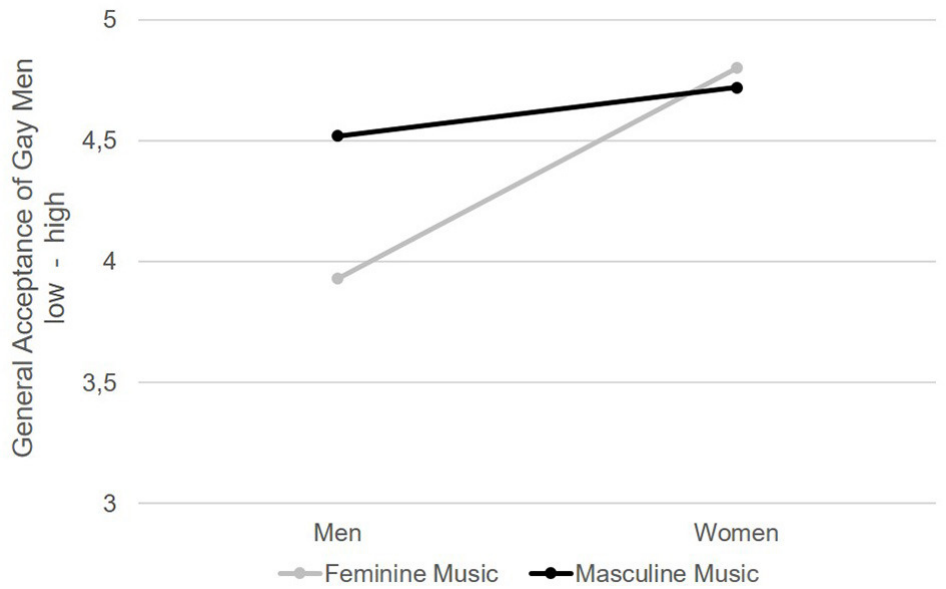
General acceptance of gay men. General acceptance of gay men was measured with four items on a five-point Likert scale, 1 = *do not agree*, 5 = *completely agree*, e.g., “I think gay men are not disgusting,” *N* = 218.

Finally, the manipulated music versions actually changed the participants' general acceptance of gay men (**H3**)—at least in the short term. As expected, overall, women were more tolerant than men—*F*_(1, 214)_ = 25.82, *p* < 0.001, η^2^ = 0.108 (see Figure 2). Although the average male participant could not be considered intolerant (*M* = 4.24, *SD* = 0.97; measures of tolerance were rated on a five-point Likert scale), those heterosexual men who saw the advertising with masculine music reported significantly more tolerance toward gay men than did those exposed to the feminine music—gender × music condition: *F*_(1, 214)_ = 10.07, *p* = 0.002, η^2^ = 0.045. Once again we found no significant main effect for music's connotation, *F*_(1, 214)_ = 5.46, *p* = 0.02, η^2^ = 0.025 (for more details, see Table 1).

## Discussion

### Conclusion

Companies with established majority-heterosexual customers continue to shy away from using gay advertising protagonists in their audio-visual commercials on television and the Internet because of heterosexual consumers' (especially men's) negative reactions to advertising campaigns targeting or featuring gay men or women (e.g., Um, 2014). The present study focused on the potential of background music in commercials—an element that is often neglected in advertising research (e.g., Shevy and Hung, 2013)—to mitigate the potential negative effects of gay male advertising protagonists. The results indicate that carefully selected feminine- and masculine-connoted music can affect the perceived gender of an advertising protagonist. The impact of this manipulation on heterosexual male recipients was especially interesting. Compared with the female participants, the male participants were generally more critical of the gay advertising protagonists. When they were exposed to masculine advertising background music, men tended to evaluate the promoted brand more positively and even had a higher general acceptance of gay men (at least in the short term), compared with men who watched the commercial with feminine music. Although the observed effects were medium or small, companies planning audio-visual advertising using gay protagonists could benefit from the purposeful use of background music.

Because there was no generally more positive effect for the more congruent, feminine music version, the previously described possible confounding effect of the differing degrees of musical fit of the music versions can probably be disregarded in interpreting the results of this study. Nevertheless, a potential competing impact of musical connotation and musical fit should be further investigated in the future.

From a managerial perspective, the study seems to indicate a tough decision. Normally, background music for audio-visual advertising should be selected as fitting to the commercial as possible because of the potential of musical fit to increase advertising efficiency (e.g., Herget et al., 2020). Based on this study's results, the feminine-connoted music that is obviously more fitting to the commercial's romantic story and is perceived as generally more congruent to the commercial has to be dismissed because of its negative effects on heterosexual male viewers. In a commercial featuring gay male protagonists, it seems wise to use the less congruent masculine-connoted music. To what extent can this recommendation be generalized? Since the perceived fit of music and commercial is influenced by many factors (Galan, 2009; Herget et al., 2018), in other commercials, masculine-connoted music could be more fitting than a feminine-connoted alternative—avoiding this tough decision. Suppose a commercial featuring gay male protagonists and addressing heterosexual males as a target group actually does call for more feminine than masculine-connoted background music. In that case, care should be taken when selecting this music version. While less congruent masculine music is recommended, previous studies suggest that entirely incongruent music has clearly negative effects on advertising parameters such as attitude toward the spot, the brand, and intentions to buy (e.g., Kellaris et al., 1993; North et al., 2004). Therefore, the perceived musical fit of the selected music versions should be at least moderate (as was the case in this study with the masculine-connoted music's perceived musical fit of *M* = 2.84*, SD* = 1.12).

### Critical Remarks and Implications for Future Research

Data collected on public opinion show a decreasing trend in homophobic ideas and mindsets (Keleher and Smith, 2012; Gong, 2020); thus, hopefully, considerations of how the use of specific background music may weaken negative reactions to gay-themed advertising will be less relevant in future research. However, while this remains a relevant topic, a few limitations of this study are worth discussing and considering in future work.

This study was based on an online-recruited convenience sample. We primarily used different social media platforms to circulate the study's invitation, resulting in a sample consisting of mostly female and young heterosexual participants with higher education. Angelini and Bradley (2010) have argued that this kind of sample is more liberal and open toward sexual and gender minorities than the general population. Larger effects might be detected with more heterogeneous samples. The uneven gender split in itself is a study limitation, confined the options to analyze the resulting data and the explanatory power of our results. By including only heterosexual participants, this study also eliminated the possibility of comparing the effects of different background music versions on participants with different sexual orientations. There was a similar problem with the stimulus: The commercial portrayed only a gay male couple. More comprehensive conclusions would be possible if commercials with gay men and lesbians could be used in future studies (Bond and Farrell, 2020b).

Although the items measuring the protagonists' perceived gender (based on Grohmann, 2009) were pretested, the scale obtained reliability of Cronbach's α = 0.67 in the main experiment. Nevertheless, based on arguments as in Kline (1993), we tested the related hypothesis. However, the results should be interpreted cautiously.

**Figure 3:**
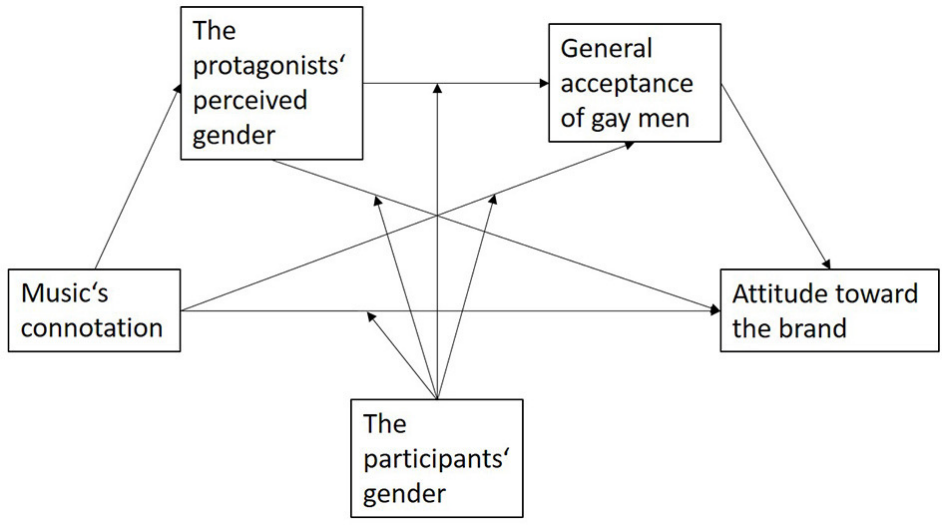
Concept of a moderated mediation model.

Another issue that should be discussed involves the different attitudes toward the brand among the participants. The present study used a jewelry commercial. It may well be that heterosexual men did not rate the brand more negatively compared with women because of the gay protagonists, but rather because heterosexual men tend to have no strong connection to jewelry. In the future, this research question should be tested with more gender-neutral brands and products (e.g., Pounders and Mabry-Flynn, 2016). It could be interesting to take a more comprehensive approach in a follow-up study measuring potential covariates, such as the tolerance of homosexuality as a trait (e.g., Um, 2014), the participant's political ideology (Northey et al., 2020), the tendency to provide socially desirable answers (e.g., Åkestam et al., 2017), or the individual likeability of the music used (e.g., Herget et al., 2020). Based on previous research and this study's results, it is reasonable to consider a moderated mediation model in which music's connotation—mediated by the protagonists' gender perception and the participants' acceptance of gay men, and moderated by the participants' gender identity (e.g., Åkestam et al., 2017)—influences brand attitude (see Figure 3). Given this study's limitations, we refrained from testing the model. In future research regarding music's impact on the perception of gay advertising protagonists, it might be worth considering it.

In this study, the higher level of tolerance of heterosexual men toward gay commercial protagonists in the condition of masculine-connoted music could be explained by a perceived similarity induced by specific background music. Masculine-connoted background music leads to a more masculine perception of gay advertising protagonists resulting in heterosexual male consumers feeling less need to distance themselves from these protagonists. Although this effect was measured in the short term, it is also reasonable to expect long-term effects (Bond and Farrell, 2020a,b). Advertising recipients are often exposed to the influence of a specific audio-visual commercial more than once (Vakratsas and Ma, 2005), which presents a logical basis for potential long-term effects. Åkestam et al. (2017) called for a more intensive exploration of the possible positive social impacts of diversity in marketing. Bearing in mind that advertising can have positive as well as negative cultural and social influences on society (e.g., Spielvogel and Terlutter, 2013; Dahlén et al., 2014; Federici and Bernardelli, 2018), recent studies have emphasized the importance of careful and conscious commercial composition (e.g., model–product fit in Pounders and Mabry-Flynn, 2016). In line with this idea, the use of specific, carefully selected background music in audio-visual advertising could at least partially prevent the unintended negative effects of gay-themed advertising.

## Data Availability Statement

The raw data supporting the conclusions of this article can be found at https://tinyurl.com/259c9xfr.

## Ethics Statement

Ethical review and approval was not required for the study on human participants in accordance with the local legislation and institutional requirements. Written informed consent was not provided because as common in online experiments on the first page of the online questionnaire the participants were asked to indicate their consent by clicking the “next”-button to start the online experiment. On this first page with the “next”-button, they were informed about the scientific background of the experiment, the anonymity of the collected data and the general procedure and duration of the following experiment. The fact that the participation in the experiment was voluntary and could be canceled at any time was also explicitly stated.

## Author Contributions

A-KH conceived the original idea. FB and A-KH developed the theoretical foundation, planned the experiment, and designed the experimental stimuli. FB carried out the experiment. A-KH wrote the manuscript. All authors contributed to the article and approved the submitted version.

## Conflict of Interest

The authors declare that the research was conducted in the absence of any commercial or financial relationships that could be construed as a potential conflict of interest.

## Footnotes

^1^In this article, the term “gay” is used in its broad interpretation, including all genders. If only men are described or addressed, the clarifying phrase “gay men” is used.

^2^Lesbian, gay, bisexual, transgender, and queer.
