# Supplementary material for: Sounds Like Respect. The Impact of Background Music on the Acceptance of Gay Men in Audio-Visual Advertising
Source: Front Psychol. 2021 Apr 22;12:645533. doi: 10.3389/fpsyg.2021.645533 (PMC8100044; doi:10.3389/fpsyg.2021.645533)
Supplement: Supplementary file 1 [file Table_1.DOCX]

**Online Questionnaires of pretest and main experiment**

Please note: Based on reliability measurements, specific items were used for the data analysis reported in the paper. These items were highlighted in bold. We also indicated with “(r)” if items were reverse coded.

**Pretest**

**1. Perception of the protagonists’ gender**

You have just seen a commercial from Tiffany & Co. Could you please judge the commercial’s protagonists based on the following attributes?

The protagonists appeared to be…

|  | I totally disagree. |  |  |  | I totally agree. |
| --- | --- | --- | --- | --- | --- |
| adventurous | ○ | ○ | ○ | ○ | ○ |
| brave | ○ | ○ | ○ | ○ | ○ |
| daring | ○ | ○ | ○ | ○ | ○ |
| dominant | ○ | ○ | ○ | ○ | ○ |
| sturdy | ○ | ○ | ○ | ○ | ○ |
| fragile (r) | ○ | ○ | ○ | ○ | ○ |
| graceful (r) | ○ | ○ | ○ | ○ | ○ |
| **sensitive (r)** | ○ | ○ | ○ | ○ | ○ |
| **sweet (r)** | ○ | ○ | ○ | ○ | ○ |
| **tender (r)** | ○ | ○ | ○ | ○ | ○ |

**2. Musical fit**

Please indicate the degree you perceived the background music used as fitting to the commercial.

|  | I totally disagree. |  |  |  | I totally agree. |
| --- | --- | --- | --- | --- | --- |
| **Regardless of how much I liked or disliked the music, it did seem appropriate for this ad.** | ○ | ○ | ○ | ○ | ○ |
| **The music did not seem to fit the message in this ad.** (r) | ○ | ○ | ○ | ○ | ○ |
| **The music was not what I would expect to hear in this kind of ad.** (r) | ○ | ○ | ○ | ○ | ○ |

**3. Socio-demographic data**

Finally, we would like to invite you to provide some personal information.

How old are you?

My age:

I’m a…

- woman
- man

What is your highest educational degree? If your degree is not listed, please select the degree that is closest to yours.

- I haven’t finished school (yet).
- I finished secondary primary school.
- I finished secondary school.
- I finished high school.
- I have got a bachelor’s/master’s degree.
- no comment

Please indicate your sexual orientation.

- heterosexual
- gay
- bisexual
- I don’t want to answer this.

**Main Experiment**

**1. Socio-demographic data**

First of all, we would like to invite you to provide some personal information.

How old are you?

My age:

I’m a…

- woman
- man

What is your highest educational degree? If your degree is not listed, please select the degree that is closest to yours.

- I haven’t finished school (yet).
- I finished secondary primary school.
- I finished secondary school.
- I finished high school.
- I have got a bachelor’s/master’s degree.
- no comment

Please indicate your sexual orientation.

- heterosexual
- gay
- bisexual
- I don’t want to answer this.

**2. Attitude toward the brand**

Thank you for watching the commercial. We are curious about your opinion on the advertised brand.

I evaluate the commercials’ brand as…

| bad | ○ | ○ | ○ | ○ | ○ | good |
| --- | --- | --- | --- | --- | --- | --- |
| unpleasant | ○ | ○ | ○ | ○ | ○ | pleasant |
| negative | ○ | ○ | ○ | ○ | ○ | positive |
| unlikeable | ○ | ○ | ○ | ○ | ○ | likable |

**3. Perception of the protagonists’ gender**

Could you please judge the commercial’s protagonists based on the following attributes?

The protagonists appeared to be…

|  | I totally disagree. |  |  |  | I totally agree. |
| --- | --- | --- | --- | --- | --- |
| **adventurous** | ○ | ○ | ○ | ○ | ○ |
| brave | ○ | ○ | ○ | ○ | ○ |
| daring | ○ | ○ | ○ | ○ | ○ |
| **dominant** | ○ | ○ | ○ | ○ | ○ |
| sturdy | ○ | ○ | ○ | ○ | ○ |
| fragile (r) | ○ | ○ | ○ | ○ | ○ |
| graceful (r) | ○ | ○ | ○ | ○ | ○ |
| **sensitive (r)** | ○ | ○ | ○ | ○ | ○ |
| sweet (r) | ○ | ○ | ○ | ○ | ○ |
| **tender (r)** | ○ | ○ | ○ | ○ | ○ |

**4. Musical fit (Cover items: fit of the actors)**

Let us focus on other specific elements that can be evaluated in a commercial. Please indicate the degree you perceived the actors and the background music used as fitting to the commercial.

|  | I totally disagree. |  |  |  | I totally agree. |
| --- | --- | --- | --- | --- | --- |
| *Regardless of how much I liked or disliked the actors, they did seem appropriate for this ad.* | ○ | ○ | ○ | ○ | ○ |
| *The actors did not seem to fit the message in this ad.* | ○ | ○ | ○ | ○ | ○ |
| *The message and the actors in the ad both seemed to evoke the same general mood.* | ○ | ○ | ○ | ○ | ○ |
| *The message and the actors in the ad both made me think about the same things.* | ○ | ○ | ○ | ○ | ○ |
| **The music did not seem to fit the message in this ad. (r)** | ○ | ○ | ○ | ○ | ○ |
| **The message and the music in the ad both made me think about the same things.** | ○ | ○ | ○ | ○ | ○ |
| **Regardless of how much I liked or disliked the music, it did seem appropriate for this ad.** | ○ | ○ | ○ | ○ | ○ |
| **The music and the message both seemed to evoke the same general mood.** | ○ | ○ | ○ | ○ | ○ |

**5. General acceptance of gay men**

Everyone can have a personal and individual opinion about gay people. Below you can see some statements about gay men. For each statement, please indicate whether you agree or disagree with it.

|  | I totally disagree. |  |  |  | I totally agree. |
| --- | --- | --- | --- | --- | --- |
| **Homosexual behavior between two men is just plain wrong. (r)** | ○ | ○ | ○ | ○ | ○ |
| I would not have a problem with gay male friends. | ○ | ○ | ○ | ○ | ○ |
| **I think gay men are disgusting. (r)** | ○ | ○ | ○ | ○ | ○ |
| If my brother told me he were gay, it wouldn’t change anything between us. | ○ | ○ | ○ | ○ | ○ |
| If a man has gay feelings, he should do everything he can to overcome them. (r) | ○ | ○ | ○ | ○ | ○ |
| Gay men fit into our society just as well as heterosexual men do. | ○ | ○ | ○ | ○ | ○ |
| **Male homosexuality is an inferior form of sexuality. (r)** | ○ | ○ | ○ | ○ | ○ |
| **The thought of gay marriage seems ridiculous to me. (r)** | ○ | ○ | ○ | ○ | ○ |

**6. Familiarity with the commercial and the background music**

|  | yes | no |
| --- | --- | --- |
| I knew this commercial. | ○ | ○ |
| I’ve heard that music before. | ○ | ○ |
